# On the Occurrence of Tachycardia in Association with Parenchymatous Goitre

**Published:** 1914-06

**Authors:** F. H. Edgeworth

**Affiliations:** Physician to the Bristol Royal Infirmary and Professor of Medicine, University of Bristol


### ON THE OCCURRENCE OF TACHYCARDIA IN ASSOCIATION WITH PARENCHYMATOUS GOITRE.

BY

F. H. Edgeworth, M.D., D.Sc.,

*Physician to the Bristol Royal Infirmary and Professor of Medicine, University of Bristol.*

---

A lady, aged 36 years, came to see me in November, 1912, and said that her thyroid gland had begun to enlarge at the age of 19 years, and that this enlargement had much increased at the age of 30, and had been accompanied by an increased rapidity of the rate of the heart. The condition had been diagnosed as one of incomplete exophthalmic goitre. Various methods of treatment, based on this diagnosis, had been adopted, but with no result.

The patient was strongly built, muscular, and weighed thirteen stone. She had a placid manner. No tremor was present, nor any exophthalmos. Both lateral lobes and the isthmus of the thyroid gland were enlarged, the right lateral lobe to a greater extent than the left. No arterial pulsation or thrill could be felt in the gland. The pulse rate was 100 per minute and regular. The systolic blood pressure was 132 mm. Hg. The apex of the heart was in the fifth space internal to the nipple line, and no evidence of enlargement was found. Its action was regular and quiet, not flapping or slapping as is usual in exophthalmic goitre; the cardiac sounds approached a tic-tac, owing to the shortness of the first sound. No symptom or physical sign of myxœdema was present.

The patient was asked to discontinue the dried pituitary gland which she had been taking, and to see me again in a month's time. It was then found that the physical signs were unchanged. The pituitary gland, apparently, had not been slowing the rate of the heart.

Dried thyroid gland was then given—one 5-grain tablet every night. At the end of three months it was found that the patient had lost a stone in weight, the size of the thyroid gland had diminished to that of a normal one, and the cardiac rate had dropped to 80 per minute. The patient said she felt quite well. The thyroid gland was then discontinued. Twelve months later the patient was still in good health, with a thyroid gland of normal size, and a pulse rate of 80.

Incomplete cases of Graves's disease come under observation from time to time—cases in which there is absence of one or more of the four cardinal signs, exophthalmos, enlargement of the thyroid gland, tachycardia, and tremor. The question which arose in my mind on first seeing the patient whose medical history is recounted above was whether she was one of these incomplete or fruste cases, or whether the tachycardia might not be in association with a simple enlargement of the thyroid. For reasons to be given later, the decision was in favour of the latter alternative, and the treatment which was correspondingly adopted was successful.

McKisack[1] gave an account of some twenty-one cases of incomplete Graves's disease, in fifteen of whom exophthalmos was absent, but in whom the cardiac rate was increased to between 110 and 130 per minute. Such cases were considered to be due to hyperthyroidism, though without exophthalmos. To one case occurring at the age of puberty he gave dried thyroid gland with the result of cure, and this case too he considered one of hyperthyroidism.

A few weeks later Berry, in his Lettsomian Lectures,[2] made the following comments on McKisack's statements: "Since it is well known that thyroid extract and iodine are harmful in true Graves's disease, even in slight cases, whereas they are very beneficial in simple parenchymatous goitre, it is difficult to understand why the writer considered these cases to be atypical Graves's disease, and not ordinary simple goitre." Berry also stated that cases of simple parenchymatous goitre with some rapidity of pulse form an extremely common type of case.

McKisack, however, only recorded one case to which this criticism applies; his other cases—as far as the evidence goes—appear to have been true cases of incomplete Graves's disease. Berry's statement, "these cases," is unnecessarily sharp, and not justified.

Berry left untouched the important question of the diagnosis between parenchymatous goitre with some tachycardia and incomplete cases of Graves's disease, and of the causation of tachycardia in the former class of case.

The features which occurred in my case suggesting the former diagnosis were the absence of emaciation, tremor and exophthalmos, the placidity of temperament, and in particular the action of the heart, the action of which was not the flapping one of Graves's disease, but a quiet tic-tac.

The causation of the tachycardia is somewhat of a

[1] *Brit. M. J.*, 1913, I., 208. [2] *Lancet*, 1913, I., 583, 668, 737.

mystery. It is well known that division of one or both of the vagi in animals increases the frequency of the cardiac rate. It is possible that a similar condition exists in man. If so, the further supposition that the enlarged thyroid presses on one or other vagus will explain the associated cardiac condition. The disappearance of the tachycardia certainly coincided with the diminution in the size of the thyroid gland, whilst the normal action of the dried thyroid the patient was taking would be to accelerate the cardiac rate.

Such tachycardia, whatever its exact cause, is very different clinically from the tachycardia of Graves's disease, whether complete or incomplete, and it is not difficult to distinguish the two conditions.

